# N-Acetylaspartate Is an Important Brain Osmolyte

**DOI:** 10.3390/biom10020286

**Published:** 2020-02-12

**Authors:** Marina Warepam, Khurshid Ahmad, Safikur Rahman, Hamidur Rahaman, Kritika Kumari, Laishram Rajendrakumar Singh

**Affiliations:** 1 Department of Biotechnology, Manipur University, Manipur 795003, India; marina_war@yahoo.in (M.W.); hamidur2006@gmail.com (H.R.); 2 Department of Medical Biotechnology, Yeungnam University, Gyeongsan, Gyeongbuk 38541, Korea; ahmadkhursheed2008@gmail.com; 3 Department of Botany, Munshi Singh College, BR Ambedkar Bihar University, Muzaffarpur, Bihar 845401, India; shafique2@gmail.com; 4 Dr. B. R. Ambedkar Center for Biomedical Research, University of Delhi, Delhi-110007, India; kritika0717@gmail.com

**Keywords:** protein stability, thermal denaturation, osmolytes, protein unfolding

## Abstract

Most of the human diseases related to various proteopathies are confined to the brain, which leads to the development of various forms of neurological disorders. The human brain consists of several osmolytic compounds, such as N-Acetylaspartate (NAA), myo-inositol (mI), glutamate (Glu), glutamine (Gln), creatine (Cr), and choline-containing compounds (Cho). Among these osmolytes, the level of NAA drastically decreases under neurological conditions, and, hence, NAA is considered to be one of the most widely accepted neuronal biomarkers in several human brain disorders. To date, no data are available regarding the effect of NAA on protein stability, and, therefore, the possible effect of NAA under proteopathic conditions has not been fully uncovered. To gain an insight into the effect of NAA on protein stability, thermal denaturation and structural measurements were carried out using two model proteins at different pH values. The results indicate that NAA increases the protein stability with an enhancement of structure formation. We also observed that the stabilizing ability of NAA decreases in a pH-dependent manner. Our study indicates that NAA is an efficient protein stabilizer at a physiological pH.

## 1. Introduction

Protein misfolding disorders have become the main cause of various neurodegenerative diseases. The most common neurodegenerative disorders are Alzheimer’s disease (AD), Parkinson’s disease (PD), and Huntington’s disease (HD). Until the 1980s, the human brain was the most ignored region of the human body. However, in the last 40 years, as neurodegenerative-related diseases have become the major focus, it has been discovered that human brain cells are highly prone to the accumulation of misfolded/aggregated proteins [1,2], as they are deficient in some of the classical anti-oxidant and chaperone systems. The brain chaperone system consists of heat shock proteins (HSP),HSP70RY, HSP70.1, HSP 60, glucose regulated protein(GRP), GRP 75, GRP 78, GRP 94and Alpha-crystallin B,. These chaperones are known to assist in modulating the apoptotic pathway and are involved in protein conformational disease pathologies. Brain cells also house a number of small molecule compounds (osmolyte/osmoprotectant) that are believed to be involved in modulating proteo-stability or proteopathic conditions. Such a molecule comprises myo-inositol, glutamate, glutamine, creatine, and choline-containing compounds (e.g., glycerol phosphorylcholine) [3,4]. Previous in vitro studies indicated that such osmolytes increase the protein stability, modulate the protein folding landscape, correct the temperature-sensitive mutant protein defect, and suppress protein aggregation [5,6,7,8,9,10,11,12].

One important molecule that neuronal cells accumulate is N-Acetylaspartate (NAA). NAA is an anionic intracellular solute that can concentrate upto 10 mM or even greater, and its level is altered in several human brain disorders, making it one of the most accepted neuronal biomarkers [13].Despite 50 years of research by neuroscientists and clinicians, the accepted role of NAA in the brain cell remains unsettled. Some of the proposed biological functions of NAA include: (i) reservoir of glutamate [14]; (ii) myelin precursor [15]; (iii) neurotransmitter and neuromodulator [16,17]; (iv) osmoregulator [18,19]; (v) neuronal dipeptide N-Acetylaspartylglutamate precursor [20]; (vi) initiating protein synthesis [21]; (vii) immune function [22]; and (viii) strong association with humans’ cognitive ability and intelligence [23]. To date, the role of NAA in protein stability (the osmoprotective front) has not been identified. Therefore, we were interested in investigating the effect of NAA on two model proteins—Ribonuclease-A (RNase-A) and lysozyme—by performing thermal denaturation and structural measurements at different pH values (ranging from pH 7.0 to 2.5). We discovered that NAA increases the protein stability in terms of both *T*_m_ and ∆*G*_D_° of the proteins at physiological pH by enhancing the structure formation. The results indicate that NAA is an important osmoprotectant and could be involved in the pathophysiology of Aβ, or alpha-synuclein-induced neurodegeneration.

## 2. Materials and Methods

### 2.1. Materials

Commercially lyophilized RNase-A (type III-A from bovine pancreas) and lysozyme (from hen egg white) were purchased from Sigma. N-Acetylaspartate (NAA) was also obtained from Sigma. Guanidinium chloride (GdmCl) was purchased from MP Biomedicals. These chemicals were used without further purification, as they were of analytical grade.

### 2.2. Methods

#### 2.2.1. Preparation of Protein Stock Solutions and Determination of Concentration

RNase-A and lysozyme solutions were dialyzed extensively against 0.1 M KCl at pH 7.0 and 4 ^o^C. The dialyzed protein stock solutions were filtered with a 0.22 μm millipore syringe filter. A single protein band during polyacrylamide gel electrophoresis was obtained for both the proteins. The concentrations of RNase-A and lysozyme solutions were determined experimentally using a molar absorption coefficient ε (M^−1^ cm^−1^) value of 9800 at 277.5 nm [24] and 39,000 at 280 nm [25], respectively. For optical measurements, all of the proteins and buffer solutions were prepared in degassed double-distilled water containing 0.1 M KCl. Different buffers were used for different pH values: 0.05 M cacodylic acid buffer for pH 7.0 to pH 5.0, 0.05 M acetate buffer for pH 4.0, and 0.05 M glycine hydrochloride buffer for pH 2.5. The pH of the protein solutions remained similar even after the addition of NAA.

#### 2.2.2. Thermal Denaturation Studies

Thermal denaturation studies of RNase-A and lysozyme were performed using a Jasco V-660 UV/Vis spectrophotometer equipped with a Peltier type controller (ETCS-761) at a heating rate of 1 °C/min from 20 °C to 85 °C. The change in absorbance for RNase-A and lysozyme was followed at 287 nm and 300 nm, respectively. Measurements were repeated three times. After each denaturation, reversibility was measured for each sample. The reversibility was checked by comparing the optical property of the native protein before and after denaturation and was found to be identical. All solution blanks were neglected during the data analysis, as they showed negligible change in absorbance with temperature. For obtaining *T*_m_ (the midpoint of heat denaturation) and ∆*H*_m_ (the enthalpy change of denaturation at *T*_m_) of each transition curve, a non-linear least-squares analysis equation was used [26]:

(1) $$y(T)=\frac{y_{N}(T)+y_{D}(T)\exp[-\Delta H_{m}/R(1/T-1/T_{m})]}{1+\exp[-\Delta H_{m}/R(1/T-1/T_{m})]}$$

where *y*(*T*) is the optical property at temperature *T* (Kelvin); *y*_N_(*T*) and *y*_D_(*T*) are the optical properties of the native and the denatured protein molecules, respectively; and R is the gas constant. During analysis, it was assumed that a parabolic function describes the dependence of the optical properties of native and denatured protein molecules: i.e., *y*_N_(*T*) = *a*_N_+*b*_N_*T*+*c*_N_*T*^2^, and *y*_D_(*T*) = *a*_D_+ *b*_D_*T +c*_D_*T*^2^, where *a*_N_, *b*_N_, *c*_N_, *a*_D_, *b*_D_, and *c*_D_ are temperature-independent coefficients. The value of ∆*C*_p_ (the constant pressure heat capacity change) was obtained from the plot of ∆*H*_m_ versus *T*_m_ at each concentration of NAA. Using values of *T*_m_, ∆*H*_m_, and ∆*C*_p_, the value of ∆*G*_D_ (*T*) (∆*G*_D_ at any temperature *T*) was estimated with the help of the Gibbs–Helmholtz equation:

(2) $$\Delta G_{D}(T)=\Delta H_{m}\left(\frac{T_{m}-T}{T_{m}}\right)-\Delta C_{p}\left[\left(T_{m}-T\right)+T\ln\left(\frac{T}{T_{m}}\right)\right].$$

#### 2.2.3. Structural Measurements

Near-UV circular dichroism (CD) spectra of the native states of RNase-A and lysozyme were measured in the presence and absence of NAA at least three times in a Jasco spectropolarimeter (J-810) equipped with a Peltier-type temperature controller (Jasco PTC-424S). A necessary subtraction for the contribution of blank from each spectrum of protein was performed. The protein concentration used was 0.5 mg/mL. The path length of the used cuvette was 10 mm. Routine calibration of the CD instrument was done with D-10-camphorsulfonic acid.

The intrinsic fluorescence of RNase-A and lysozyme was measured in a Perkin–Elmer-LS 55 at least three times in the absence and presence of NAA. The protein concentration used was 3 µM. The excitation wavelength was 268 nm for RNase-A and 280 nm for lysozyme. A necessary blank subtraction was made.

## 3. Results

**Figure 1:**
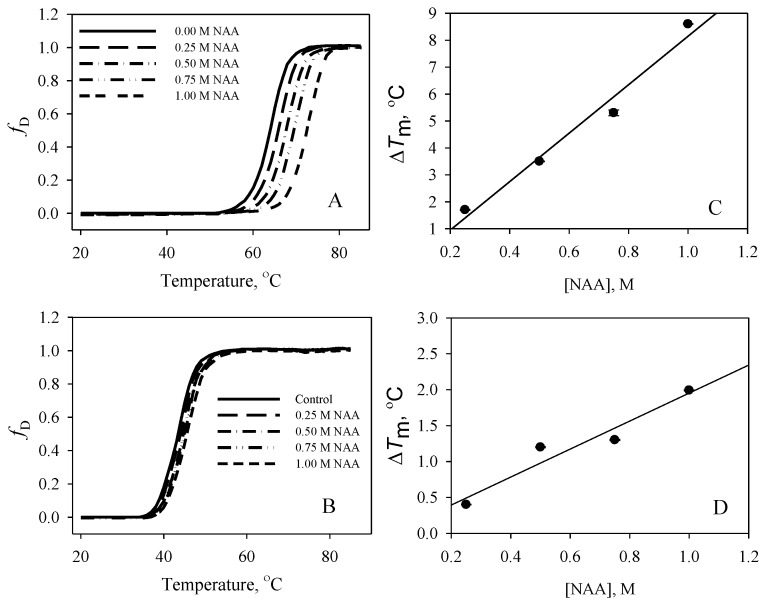
Thermal denaturation profiles of Ribonuclease-A (RNase-A) at pH 7.0 and pH 2.5. Left panels: Denaturation curves of RNase-A at pH 7.0 (**A**) and at pH 2.5 (**B**) in the absence and presence of various N-Acetylaspartate (NAA) concentrations. Right panels: Plots of ∆*T*_m_ versus NAA of RNase-A at pH 7.0 (**C**) and at pH 2.5 (**D**).The protein concentration used was 31µM.

**Figure 2:**
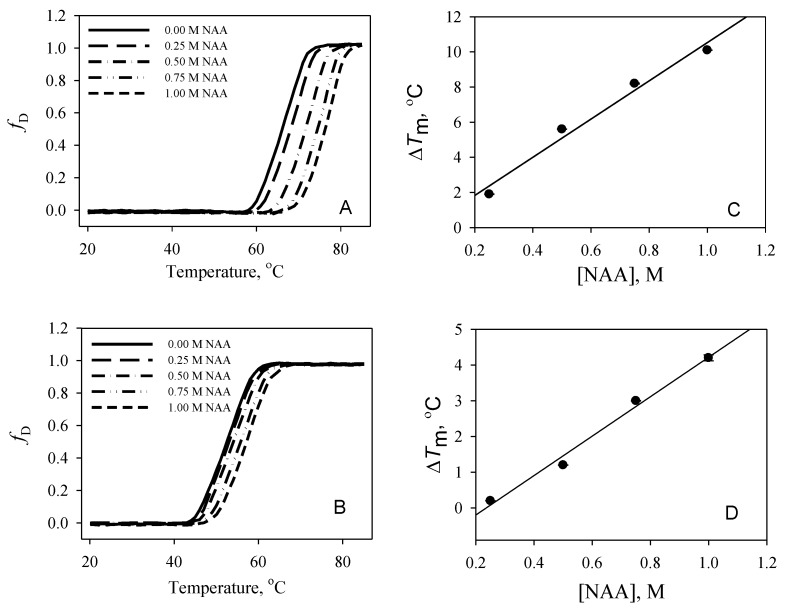
Thermal denaturation profiles of lysozyme at pH 7.0 and pH 2.5. Left panels: Denaturation curves of lysozyme at pH 7.0 (**A**) and at pH 2.5 (**B**) in the absence and presence of various NAA concentrations. Right panels: Plots of ∆*T*_m_ versus NAA of lysozyme at pH 7.0 (**C**) and at pH 2.5 (**D**).The protein concentration used was 58 µM.

To investigate the effect of NAA on protein stability, the thermal denaturation of the proteins in the absence and presence of various concentrations of NAA at each pH value was monitored by observing changes in ∆*A*_λ_ (the difference in absorbance at the wavelength, λ); ∆*A*_287_ nm for RNase-A; and ∆*A*_300_ nm for lysozyme. The denaturation of each protein was reversible at all pH values for the entire range of the molar concentration of NAA. Figure 1 and Figure 2 show the denaturation curves of RNase-A and lysozyme, respectively, in the absence and presence of different NAA concentrations (0.25 M, 0.50 M, 0.75 M, and 1.00 M) at pH 7.0 and pH 2.5.The denaturation curves are shown as a function of *f*_D_ versus temperature. *f*_D_ represents the denatured fraction of the optical properties of native and denatured protein molecules, and is obtained using the relation:

*f*_D_ = (*y* − *y*_n_)/(*y*_d_ − *y*_n_) (3)

where *y* is the optical property, and *y*_n_ and *y*_d_ are the optical properties of the native and the denatured protein molecules, respectively. Each thermal denaturation curve was analyzed for *T*_m_ and ∆*H*_m_ using Equation (1). Table 1 shows the value of *T*_m_ and ∆*H*_m_ of the proteins in the presence and absence of various concentrations of NAA at all of the pH values. It should be noted that a complete denaturation curve of lysozyme could not be obtained in the temperature range (from 20 °C to 85 °C) at pH 7.0, pH 5.5, and pH 5.0 values. To obtain a complete denaturation curve, the thermal denaturation temperature was brought down into a measurable temperature range by adding 1.5 M GdmCl to each sample. To maintain uniformity, we also added 1.5 M GdmCl to all protein samples below pH 5.0. The right panels in Figure 1 and Figure 2 also show the effect of different concentrations of NAA on the *T*_m_ of the proteins. Using the measured values of ∆*H*_m_ and *T*_m_ obtained at all of the pH values, ∆*C*_p_ values at each of the NAA concentrations were estimated from the plots of ∆*H*_m_ versus *T*_m_ (data not shown). The values of ∆*C*_p_ estimated in this manner are given in Table 1. Using the measured values of *T*_m_, ∆*H*_m_, and ∆*C*_p_, ∆*G*_D_° values (∆*G*_D_ value at 25 °C) of all of the proteins in the presence and absence of each concentration of NAA were estimated using Equation (2). However, a large extrapolation is required in such types of estimation, which increases the chance of a large error in the ∆*G*_D_° determination due to errors in the estimations of *T*_m_, ∆*H*_m_, and ∆*C*_p_. Therefore, we determined the maximum and minimum errors associated with the ∆*G*_D_° determination in a given solvent condition using Becktel and Schellman’s procedure [27]. We obtained six values of ∆*G*_D_° (three maximum and three minimum values), as there were three independent measurements of *T*_m_ and ∆*H*_m_ of a protein at a given pH and NAA concentration. Thus, the obtained six values were used to determine the average ∆*G*_D_° and the mean error. The mean error associated with the ∆*G*_D_° estimation was in the range 6–9% for both of the proteins. Table 1 shows the average values of ∆*G*_D_°. It can be seen in the table that *T*_m_ and ∆*G*_D_° of RNase-A and lysozyme increase at all of the pH values in the presence of NAA. The results indicate that NAA is a protein stabilizer.

**Figure 3:**
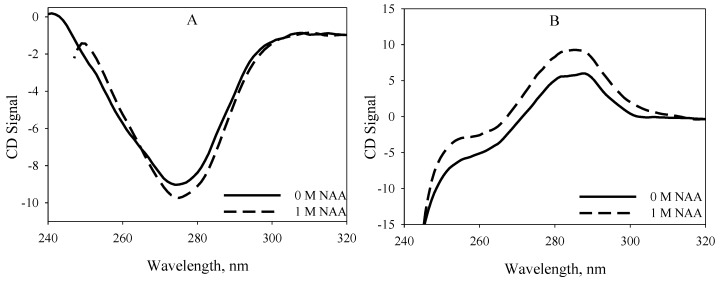
Effect of NAA on the tertiary structure of native proteins at 25 °C. Near-UV CD spectra of the native states of RNase-A (**A**) and lysozyme (**B**) in the absence and presence of NAA at pH 7.0.

**Figure 4:**
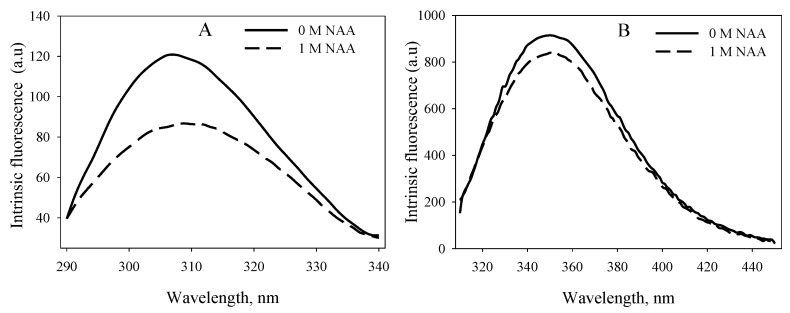
Effect of NAA on the intrinsic fluorescence of native proteins at 25 °C.Tyrosine fluorescence spectra of RNase-A (**A**) and tryptophan fluorescence spectra of lysozyme (**B**) in the absence and presence of NAA at pH 7.0.The protein concentration used was 3 µM.

We further investigated whether NAA-induced stabilization would bring about conformational alteration on the native state of the proteins. For this, we measured the near-UV CD spectra of the proteins at pH 7.0 (Figure 3). Far-UV CD spectra could not be measured due to high voltage in the presence of NAA. Figure 3 shows an alteration in the tertiary structure of the native proteins, in which there is a significant increase in the CD signal of RNase-A towards a more negative signal and lysozyme towards a more positive signal in the presence of 1M NAA. Such a change in the conformation of native RNase-A and lysozyme was also observed in the tyrosine and tryptophan fluorescence, where the fluorescence intensity of the proteins in the presence of 1M NAA decreased from native protein’s fluorescence intensity (see Figure 4). From Figure 3 and Figure 4, it appears that there is a gain in the tertiary structures of native RNase-A and lysozyme due to NAA, indicating structural stabilization/formation by NAA. Thus, the findings indicate that the increase in thermodynamic stability of the proteins is due to their structural enhancement by NAA. The observation on structural alteration by NAA is in agreement with other studies that potent protein stabilizers induce protein stability by a gain in the structure [28,29].

**Figure 5:**
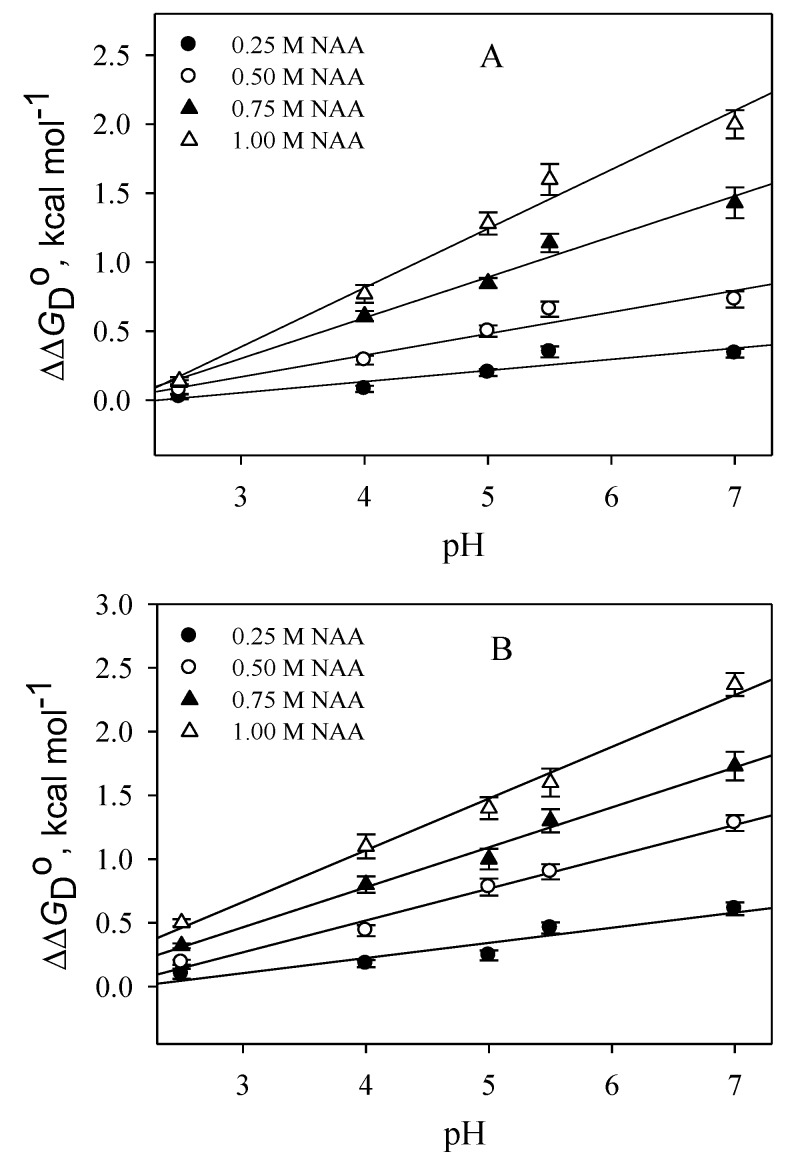
Plots of ∆∆*G*_D_° versus pH. (**A**) RNase-A (**B**) Lysozyme. The error shown here is from three independent measurements.

We were further interested in investigating the pH-dependent behavior of protein stabilization by NAA. As shown in Table 1, the stabilizing effect of NAA on the protein is higher at pH 7.0, but is highly reduced at the lowest pH values. To further confirm the pH dependence of the effect of NAA on protein stability, we have plotted ∆∆*G*_D_° (∆*G*_D_° of protein in the presence of NAA—∆*G*_D_° of protein in the absence of NAA) as a function of pH (see Figure 5). It was observed in the figure that the dependence of ∆∆*G*_D_° on pH is linear at each NAA concentration. The results indicate that the interaction of the NAA with the protein might be different at different pH values.

## 4. Discussion

**Figure 6:**
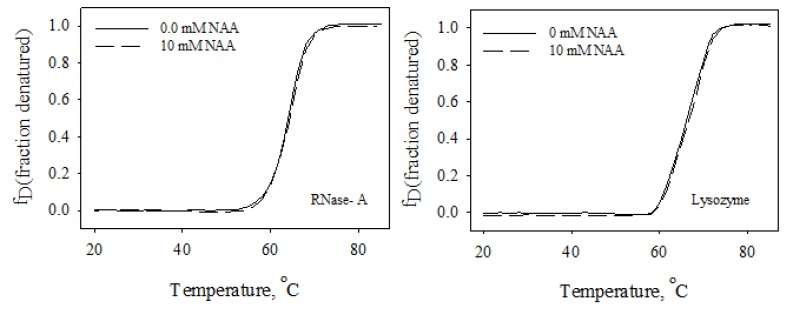
Thermal denaturation profiles of RNase-A and lysozyme at pH 7.0 in the presence of a physiological concentration of NAA.

Osmolytes are known to be responsible for the maintenance of the osmotic balance of cells under harsh stresses. In addition to maintaining the osmotic balance, osmolytes also protect the functional integrity of the macromolecules from the denaturing action of stresses by increasing the thermodynamic stability of the proteins, thereby protecting the macromolecules from stresses. Although many of the brain’s osmoprotectants have been investigated for their structural and thermodynamic consequences, the effect of NAA has never been elucidated. The present study, therefore, was carried out to investigate the function of NAA against heat stress on macromolecules. Our results (see Table 1 and Figure 1 and Figure 2) indicate that NAA increases the thermodynamic stability of the proteins, and, hence, might be a stabilizer of important proteins in neuronal cells. We have also observed that the increase in thermodynamic stability of the proteins is due to increased tertiary level interactions (Figure 3 and Figure 4). We also investigated whether the physiological NAA concentration (10 mM) could also bring about the stabilization of the proteins by undergoing similar measurements (Figure 6). As is evident in Figure 6, we could not observe any noticeable increase in the *T*_m_ of the proteins, indicating that the physiological NAA concentration is insufficient to induce the thermodynamic stabilization of proteins. However, we cannot rule out the possibility of its having a significant effect in the neuronal cells because the intracellular environment is highly crowded as compared to the dilute environment [30,31]. To bring about a change in the structure of water, it might require very minimal concentration, as opposed to the in vitro dilute buffers. This argument, however, requires experimental justification.

It is generally believed that osmoprotectants modulate the thermodynamic stability of proteins by affecting the denaturation equilibrium, N state ↔ D state. Therefore, the observed effect on the increase ∆*G*_D_° of the proteins is due to the shift in the denaturation equilibrium, N state ↔ D state towards the left. At present, the study lacks a finding of any concrete molecular-level interaction between proteins and NAA to explain the observed phenomenon: increased thermodynamic stability in the presence of NAA at pH 7.0. However, the results clearly indicate that NAA has chaperonic activity to protect proteins from temperature stress. In agreement, several osmolytic compounds present in the neuronal cells and other tissues have already been reported to have the ability to protect proteins from stress by increasing *T*_m_ or ∆*G*_D_° [12,32]. However, most of these osmolytic compounds, as invoked by Yancey (1982), should be neutral (or uncharged), as charged osmolytes (e.g., arginine and histidine) often destabilize proteins and enzymes. NAA, an amino acid derivative with a methyl group, exists as an anion with two negative charges at physiological pH [33]. Therefore, the use of NAA as an osmolytic agent might not be evolutionarily favored in nature. Speculatively, the majority of the cells might not have evolved to use NAA as an osmolytic agent, which could be the reason why NAA is not cosmopolitan in distribution, but mainly confined to brain cells only.

NAA is a molecule whose levels are altered under different pathophysiologies, including Alzheimer’s disease (AD), Huntington’s disease (HD), and Canavan’s diseases [13]. Our results imply that, inside the intracellular environment, NAA should be responsible for the protection of many important proteins involved in the brain pathophysiologies. For instance, Aβ accumulations in the brain cells are considered to be the hallmark for the pathogenesis of AD [34]. In this regard, a previous study demonstrated that NAA inhibits the initiation of Aβ fibril formation [35]. Our results further corroborate that the inhibition of the Aβ aggregation by NAA might be due to changes in the Aβ conformation (due to NAA) to a non-amyloidogenic form and the subsequent stabilization of this species as against the (aggregation-prone) amyloidogenic conformer. Thus, increased production and accumulation of NAA in brain cells would be a viable strategy toward a therapeutic intervention for AD. The effect of NAA on other important diseases associated with proteins should be investigated in the future.

Many osmolytes have previously been reported to have pH-dependent protein stabilization [36,37]. For instance, TMAO (Trimethylamine N-oxide)destabilizes proteins at low pH values, while the polyols’ ability to increase protein stabilization is greater at lower pH values as compared to physiological pH. It is evident from the results (Figure 5) that there is a pH-dependent stabilization effect of NAA on both the proteins, which is most probably due to alterations in the micro-environment of the interacting groups in NAA by pH. Thus, variation in the pH in the neuronal cells might play a crucial role in the NAA–protein interaction, leading to different effects on macromolecules. Interestingly, it has been recently reported that the pH in the neuronal cells is more variable than previously believed [38]. Future studies into understanding the physiological role of NAA in different pH conditions need to be focused on.

## Figures and Tables

**Table 1:** Thermodynamic parameters of RNase-A and lysozyme in the presence of different concentrations of NAA ^a,b,c^.

**RNase-A**																
	**pH 7.0**			**pH 5.5**			**pH 5.0**			**pH 4.0**			**pH 2.5**			
**[NAA]**	***T*_m_**	**∆*H*_m_**	**∆*G*_D_°**	***T*_m_**	**∆*H*_m_**	**∆*G*_D_°**	***T*_m_**	**∆*H*_m_**	**∆*G*_D_°**	***T*_m_**	**∆*H*_m_**	**∆*G*_D_°**	***T*_m_**	**∆*H*_m_**	**∆*G*_D_°**	**∆*C*p**
0.00	64.1	111	9.9	60.8	106	8.9	60.0	106	8.8	57.2	105	8.2	43.6	85	4.3	1.25
0.25	65.8	113	10.2	62.5	109	9.3	61.0	107	9.0	57.7	107	8.3	44.0	84	4.3	1.33
0.50	67.6	116	10.6	63.9	113	9.7	62.2	111	9.3	58.7	108	8.5	44.8	85	4.4	1.40
0.75	69.4	121	11.3	65.6	116	10.1	64.0	113	9.6	60.5	110	8.8	44.9	85	4.4	1.47
1.00	72.7	124	11.9	67.5	118	10.5	65.4	116	10.1	61.3	111	9.0	45.6	84	4.4	1.50
	(64.4)															
**Lysozyme**																
	**pH 7.0**			**pH 5.5**			**pH 5.0**			**pH 4.0**			**pH 2.5**			
**[NAA]**	***T*_m_**	**∆*H*_m_**	**∆*G*_D_°**	***T*_m_**	**∆*H*_m_**	**∆*G*_D_°**	***T*_m_**	**∆*H*_m_**	**∆*G*_D_°**	***T*_m_**	**∆*H*_m_**	**∆*G*_D_°**	***T*_m_**	**∆*H*_m_**	**∆*G*_D_°**	**∆*C*p**
0.00	66.4	106	8.6	64.1	99	7.5	63.2	96	71	61.3	91	6.5	52.8	82	4.9	1.65
0.25	68.3	111	9.2	65.7	103	8.0	64.1	98	7.3	62.4	94	6.7	53.0	83	5.0	1.71
0.50	72.0	116	9.9	67.4	107	8.4	66.2	103	7.9	63.9	96	6.9	54.0	84	5.1	1.77
0.75	74.6	121	10.3	70.6	111	8.8	69.0	106	8.1	66.4	100	7.3	55.8	85	5.2	1.86
1.00	76.5 (66.8)	127	11.0	72.9	114	9.1	71.2	110	8.5	68.8	103	7.6	57.0	87	5.4	1.92

^a^ Errors in *T*_m_, ∆*H*_m_, ∆*G*_D_°, and ∆*C*_p_ from triplicate measurements are 0.1–0.6%, 2–5%, 6–9%, and 5–7%,respectively. ^b^ Units of *T*_m_, ∆*H*_m_, ∆*G*_D_°, and ∆*C*_p_ are ^o^C, kcal mol^−1^, kcal mol^−1^,and kcal mol^−1^K^−1^, respectively. ^c^Values in parenthesis were obtained at the physiological concentration10 mM of NAA.
